# Using a trauma-informed policy approach to create a resilient urban food system

**DOI:** 10.1017/S1368980018000198

**Published:** 2018-02-20

**Authors:** Amelie A Hecht, Erin Biehl, Sarah Buzogany, Roni A Neff

**Affiliations:** 1 Department of Health Policy and Management, Johns Hopkins Bloomberg School of Public Health, Baltimore, MD, USA; 2 Center for a Livable Future, Johns Hopkins Bloomberg School of Public Health, 615 N. Wolfe Street, W7010, Baltimore, MD 21205, USA; 3 Department of Environmental Health & Engineering, Johns Hopkins Bloomberg School of Public Health, Baltimore, MD, USA; 4 Baltimore Food Policy Initiative, Baltimore Office of Sustainability, Baltimore, MD, USA

**Keywords:** Food security, Resilience, Policy, Trauma

## Abstract

**Objective:**

Food insecurity is associated with toxic stress and adverse long-term physical and mental health outcomes. It can be experienced chronically and also triggered or exacerbated by natural and human-made hazards that destabilize the food system. The *Baltimore Food System Resilience Advisory Report* was created to strengthen the resilience of the city’s food system and improve short- and long-term food security. Recognizing food insecurity as a form of trauma, the report was developed using the principles of trauma-informed social policy. In the present paper, we examine how the report applied trauma-informed principles to policy development, discuss the challenges and benefits of using a trauma-informed approach, and provide recommendations for others seeking to create trauma-informed food policy.

**Design:**

Report recommendations were developed based on: semi-structured interviews with food system stakeholders; input from community members at outreach events; a literature review; Geographic Information System mapping; and other analyses. The present paper explores findings from the stakeholder interviews.

**Setting:**

Baltimore, Maryland, USA.

**Subjects:**

Baltimore food system stakeholders stratified by two informant categories: organizations focused on promoting food access (*n* 13) and community leaders (*n* 12).

**Results:**

Stakeholder interviews informed the recommendations included in the report and supported the idea that chronic and acute food insecurity are experienced as trauma in the Baltimore community.

**Conclusions:**

Applying a trauma-informed approach to the development of the *Baltimore Food System Resilience Advisory Report* contributed to policy recommendations that were community-informed and designed to lessen the traumatic impact of food insecurity.

Food insecurity, defined as limited or uncertain access to adequate food^(^ ^1^ ^)^, creates challenges far beyond the direct effects of inadequate nutrition. Emerging evidence links food insecurity with ‘toxic stress’, or overwhelming stress associated with economic deprivation and other forms of adversity that can cause long-term physical and emotional harm^(^ ^2^ ^–^ ^4^ ^)^. It is easy to imagine how food insecurity causes lasting damage when considering the trade-offs individuals and families are forced to make between food and other necessary expenses such as asthma inhalers or monthly utility bills^(^ ^3^ ^)^. Some parents even make the decision to feed their children and go hungry themselves^(^ ^3^ ^,^ ^5^ ^)^. Despite parental attempts to shield them, children in food-insecure households often experience physical awareness (hunger, pain, tiredness, weakness), emotional awareness (worry, sadness, anger) and cognitive awareness (knowing that food is running low or of poor quality)^(^ ^6^ ^,^ ^7^ ^)^.

Food-insecure adults and children experience increased rates of mental health issues including depression, anxiety and post-traumatic stress disorder^(^ ^8^ ^–^ ^10^ ^)^. A growing body of evidence links food insecurity with risk of adverse childhood experiences and exposure to violence^(^ ^11^ ^–^ ^13^ ^)^. Food insecurity is also associated with risks for chronic disease among adults^(^ ^14^ ^–^ ^16^ ^)^ and with poor physical health and developmental delay among children^(^ ^17^ ^–^ ^19^ ^)^.

Trauma is broadly defined as an experience that is emotionally painful, distressful or shocking, and often results in long-term mental and physical health consequences^(^ ^20^ ^)^. Trauma can occur from singular incidents or repeated or prolonged exposures to negative conditions^(^ ^21^ ^)^. Given the painful and distressing nature of food insecurity and the physical and mental health consequences that often accompany it, we argue that, for many, food insecurity creates trauma. Food insecurity is often also accompanied by other poverty-related stressors that worsen the traumatic impact^(^ ^12^ ^,^ ^22^ ^)^. Additionally, individuals experiencing food insecurity may engage in risky behaviours to meet their needs (e.g. stealing, trading sex for money to buy food), which could increase the potential for trauma^(^ ^23^ ^,^ ^24^ ^)^. Accordingly, solutions to address food insecurity should employ a trauma-informed approach.

Food insecurity can be triggered or exacerbated by natural and human-made hazards that destabilize the local, regional or global food system, such as climate change-associated extreme weather events or social unrest. Recovering from such events and preventing escalated food insecurity requires strong pre-event food system functioning and advanced planning.

Baltimore, Maryland is an example of a city that is developing policies to both reduce the trauma of chronic food insecurity and minimize the effects of disruptive events on food security. Relative to the rest of the USA, rates of food insecurity are disproportionately high in Baltimore: 24 % of Baltimore households report experiencing food insecurity compared with 13 % nationally^(^ ^25^ ^,^ ^26^ ^)^. Moreover, one in four Baltimore households is located in a food desert, which the city defines as an area where the distance to a supermarket is more than 0·25 mile (0·4 km), median household income is at or below 185 % of the Federal Poverty Level, over 30 % of households have no vehicle access and the average Healthy Food Availability Index score for all food stores is low^(^ ^27^ ^)^. In Baltimore, one in three school-aged children, one in four seniors and one in three African-Americans live in food deserts^(^ ^27^ ^)^.

In 2013, the Baltimore Office of Sustainability developed a disaster preparedness plan for the city^(^ ^28^ ^)^. The plan largely excluded food, recommending instead that the city draft a separate plan focused on food system resilience^(^ ^28^ ^)^. Following the death of Freddie Gray from injuries sustained while in police custody in April 2015, some demonstrations in Baltimore gave way to looting, destruction of property and arson^(^ ^29^ ^)^. The uprisings affected food security in parts of the city – several food retail outlets were looted or damaged, public schools (which provide school meals) were closed and the Mayor imposed a temporary overnight curfew that disrupted food deliveries^(^ ^29^ ^)^. The event forced officials to recognize that the city was unprepared to deal with crises that threaten the food system.

As a result of these events, in 2016, the Baltimore Food Policy Initiative, an intergovernmental collaborative housed in the Baltimore Office of Sustainability and created to address food access in Baltimore’s food deserts^(^ ^30^ ^)^, began to develop a plan for a more resilient urban food system – one able to adapt to local and global challenges posed by climate change, political and economic crises, natural disasters and other factors (broadly encompassed by the term ‘hazards’).

To inform the plan, food policy planners at the Baltimore Food Policy Initiative, food systems researchers with expertise in policy at the Johns Hopkins Center for a Livable Future, and other experts in public health, health security, community development and sustainability worked together to develop the *Baltimore Food System Resilience Advisory Report* ^(^ ^31^ ^)^. All authors of the present paper were co-authors or reviewers involved in developing the report.

The report will inform the city’s final Food System Resilience Plan and includes an assessment of the current food system, threats the food system is most likely to encounter, the populations, infrastructure and assets most vulnerable to those hazards, as well as recommendations for how to improve system resilience. Specifically, policy recommendations aim to increase food security both on a day-to-day basis and in the face of hazards to the food system, with a special focus on vulnerable populations.

To inform report development, we used Bowen and Murshid’s framework for trauma-informed social policy^(^ ^32^ ^)^. The framework applies the principles of trauma-informed practice – a model of service provision used across a variety of health and social service settings to address consequences of trauma, facilitate healing and prevent re-traumatization^(^ ^33^ ^)^ – to the policy formulation process^(^ ^32^ ^)^. The authors argue for moving ‘beyond broad notions of trauma as a universal experience [to] address its specific sociopolitical and economic roots as well as its disproportionate impacts among marginalized populations’^(^ ^32^ ^)^. The framework builds on existing frameworks for policy development and analysis, including Rapp *et al*.’s strengths-based social policy analysis model^(^ ^33^ ^)^, and incorporates elements that make it uniquely suited to address issues related to trauma^(^ ^32^ ^)^. Specifically, the framework focuses on the six core principles of trauma-informed practice: safety; trustworthiness and transparency; collaboration and peer support; empowerment; choice; and the intersectionality of identity characteristics (Table 1)^(^ ^32^ ^)^.

**Table 1:** Bowen and Murshid’s six core principles of trauma-informed social policy^(^ ^32^ ^)^

Principle	Definition
Safety	Ensure physical and emotional safety; prevent further trauma from occurring
Trustworthiness and transparency	Maintain transparency in policies and procedures, with the objective of building trust among stakeholders
Collaboration	View policy’s target population as active partners in policy development and implementation and as experts in their own lives
Empowerment	Share power with policy’s target population, giving them a strong voice in decision making
Choice	Preserve meaningful choices for policy’s target population to maintain a sense of control
Intersectionality	Focus on awareness of identity characteristics and the privileges or oppression these characteristics can incur

The *Baltimore Food System Resilience Advisory Report* seeks to achieve the four R’s of the trauma-informed approach^(^ ^34^ ^)^: it realizes the widespread impact of trauma associated with food insecurity, recognizes the signs of trauma in community residents, and seeks to respond by integrating knowledge about trauma into policies and practices to prevent food insecurity that actively resist re-traumatization.

Interest in creating policy that incorporates trauma-informed practice has grown in recent years^(^ ^35^ ^,^ ^36^ ^)^. In Baltimore, the City Health Department’s 2020 strategic blueprint incorporates a trauma-informed approach as a core value guiding planning efforts^(^ ^37^ ^)^. At the national level, from 2009 to 2015, forty-nine bills were introduced in the US Congress explicitly mentioning trauma-informed practice^(^ ^38^ ^)^. Yet to our knowledge, no study in the published literature has focused on the process of applying the principles of trauma-informed social policy to the policy development process. In the present paper, we provide a real-world example of applying the framework to policy formulation, discuss limitations of the approach and provide recommendations for others seeking to use a trauma-informed approach to the development of food policy.

## Methods

The policies and strategies recommended in the *Baltimore Food System Resilience Advisory Report* were developed based on: input from stakeholder interviews; conversations about food access with community members at community outreach events; input from participants in the Baltimore City Emergency Food Working Group and the Baltimore City Food Policy Action Coalition; a literature review encompassing food system plans from other cities and evidence on preparedness, resilience and climate change; and mapping that identified areas of vulnerable populations and food system infrastructure. The present paper reports and discusses findings from the stakeholder interviews.

### Recruitment and sampling

We performed semi-structured in-depth interviews (*n* 25) with Baltimore food system stakeholders stratified by two informant categories: thirteen organizations focused on promoting food access, such as governmental offices and non-profits; and twelve community leaders from predominantly low-income and African-American neighbourhoods. Participants were selected purposively based on recommendations from project partners and other colleagues who work with a range of organizations and community leaders in the city, as well as through snowball sampling, until data saturation was reached.

Organizations promoting food access were selected with the aim of identifying a diversity of non-profit and governmental organizations that play a role in increasing access to food in Baltimore. These included food pantries, soup kitchens, food rescue organizations, disaster relief organizations, out-of-school meal providers, meal delivery organizations, social service referral providers and government food assistance programmes. At each organization, we interviewed the person most directly involved in the organization’s emergency planning efforts, commonly the leader of the organization or programme.

We defined ‘community leader’ as the head of a neighbourhood association or church (institutions that serve as important centres of community life in Baltimore) who had observed how recent events such as the Baltimore Uprising in April 2015 and Winter Storm Jonas in January 2016 had impacted food security in their community. To understand how food access challenges and community responses differed across the city, we sought geographic diversity among community leaders. Participants were eligible for the study if they were aged ≥18 years and lived or worked in Baltimore City.

### Data collection

Semi-structured interviews were conducted from June to September 2016. Interviews covered topics including experiences in past events that disrupted food access in Baltimore City, preparation methods undertaken to mitigate effects of hazards and ways in which Baltimore City can support resumption of normal activity following an event in the future. Interviews took place in person or by telephone and lasted 30–55 min. All participants provided informed verbal consent. Recordings were transcribed and identifying information was redacted prior to analysis. Participants received $US 25 gift cards. The study was approved by the Johns Hopkins Bloomberg School of Public Health Institutional Review Board (IRB No. 7169).

### Data analysis

Data were managed and analysed using ATLAS.ti version 6. Using a phronetic iterative approach^(^ ^39^ ^)^, the research team developed an analytic codebook composed of ten coding families and 167 codes. Three researchers coded transcripts, meeting regularly to discuss findings and reconcile differences. After coding, data were extracted and analysed. Relevant codes were categorized into five major themes: (i) trauma; (ii) food insecurity; (iii) community cohesion; (iv) food choice; and (v) recommendations for the city.

Ellipses (…) denote minor deletions that do not affect meaning.

## Results

In this section, we first summarize the perspectives of community leaders (CL) and food access organization (FAO) representatives on the relationship between food insecurity and trauma in Baltimore. We then describe how the principles of the trauma-informed framework were interpreted and applied to the policy formulation process, incorporating comments from interviewees.

### Link between food insecurity and trauma

Interviews with community leaders suggested that food insecurity is experienced as trauma. Community leaders discussed trauma induced by both chronic and acute food insecurity.

Participants described seeing neighbours and friends struggle to find food on a regular basis, a challenge often complicated by other issues in the home or neighbourhood, such as violence:

‘It’s just daily, it’s just every day. And then with trauma stuff, your bandwidth is just so narrow, when you experience violence. You know, your focus becomes very singular and lots of times it is where you’re like: “How am I going to feed my kids? How am I going to handle this? What am I going to do?”’ (CL1)

When asked about steps the city can take to ensure adequate food access in emergencies, some participants grew frustrated, explaining that chronic food insecurity must be addressed before the city can consider planning for emergencies. The same community leader explained:

‘It’s really an issue the families are dealing with regardless of the weather. Certainly, some are impacted more with weather, but it’s just ongoing … I don’t know what the solution is, but I don’t think it’s … seasonal. It isn’t event-oriented. It’s a chronic issue. Maybe it’s just too much for us to hold on to, to realize there’s that much hunger out there.’ (CL1)

Community leaders discussed how some Baltimore residents, particularly people of colour, feel they are in crisis every day. One community faith leader explained the mechanisms already in place in communities to support residents with the daily challenge of finding enough to eat:

‘I would even say that in a Black community … and in other communities of colour, and in poor communities, a lot of times we already in states of emergency before the weather happens so we figure out ways to be resourceful and to lean on one another, to ensure that the community is fed, even typically speaking.’ (CL2)

Community leaders also discussed acute food insecurity precipitated by specific events such as snowstorms, power outages and civil unrest. For example, one community leader said some residents were left without access to basic necessities following the Baltimore Uprising:

‘They had nowhere to go. Especially the older people. They couldn’t get their medication … and simple things like bread and milk. They had nowhere to go.’ (CL3)

Residents who are elderly, disabled and homebound are particularly vulnerable to acute food insecurity during weather-related events. One interviewee described a homebound resident who was stranded without food when his regular home food delivery service was unable to reach him during a major snowstorm:

‘We had a gentleman who had not eaten in several days and he was trapped in his house and he was homebound and there was so much snow and he couldn’t get out ...’ (FAO1)

The speed at which the city responds to crises (by clearing roads and reopening public transportation, for example) can determine how many and to what degree people are impacted. Several community leaders explained that in the past, the city has responded slowly to crises such as snowstorms, taking several days to plough major roads. One employee of a food assistance organization described that after the last major snowstorm, it was a week before the food system was operating normally:

‘I think it really depends on the city’s response time. I think that’s the biggest hurdle because once streets are clear, so deliveries are able to come … people feel safer about coming out.’ (FAO2)

These quotes provide anecdotal evidence that food security is experienced in Baltimore as both an acute and chronic trauma, and that the city’s response to crises that threaten the food system impacts how and by whom food security is experienced.

### Applying the trauma-informed framework

To address both chronic and acute food insecurity, the report incorporates recommendations informed by the six core principles of trauma-informed policy (Table 2). In this section, we explain how each principle was interpreted and applied.

**Table 2:** Principles of the trauma-informed social policy framework addressed by selected policy recommendations in the *Baltimore Food System Resilience Advisory Report* ^(^ ^31^ ^)^

Example policy recommendation	Safety	Trustworthiness and transparency	Collaboration and peer support	Empowerment	Choice	Intersectionality
Enhance capacity of food assistance organizations to provide for clients’ special dietary needs	X		X	X	X	X
Work with community members to develop neighbourhood-specific food storage plans and ensure that food stored is culturally appropriate, safely used and anticipates special dietary needs of community members	X	X	X	X	X	X
Continue to actively solicit input from diverse members of the community, including those who do not typically attend community meetings		X	X	X		
Identify ways to support community-based organizations (e.g. providing or identifying financial resources and technical support) to enhance their ongoing work to reduce food insecurity as well as preparedness efforts		X	X	X		
Continue to advocate for policies and programmes that reduce food insecurity by addressing its root causes, including poverty, employment and discrimination				X		X
Incentivize food retailers to strengthen backup systems and equipment (e.g. cyber/data backups, insurance, generators, energy-efficient refrigeration, solar power)	X				X	
Support community-led efforts to engage in urban farming and community gardening			X	X	X	X
Support community-owned business development, particularly minority-owned new business development in the food sector			X	X	X	X

#### Safety

Trauma-informed policy should focus on ensuring the basic safety (e.g. food security) of vulnerable populations and avoid privileging the safety of one group at the expense of another^(^ ^32^ ^)^. Based on prior knowledge of the research team, mapping and interviews, we identified populations in the city most vulnerable to chronic and/or acute food insecurity: children, older adults, people living in food deserts, people of low and marginal income, people with disabilities, people who are homebound, people experiencing homelessness and people with special dietary needs.

The report focuses on the distinct threats these populations face in accessing nutritious and affordable food in times of crisis and recommends strategies to reduce their exposure both on a day-to-day basis and in crisis. For example, people who live in food deserts and lack reliable transportation may not have regular access to healthy food retailers. When adverse weather events such as snowstorms occur, barriers to access may be further exacerbated by unploughed roads and stalled public transportation. To keep these populations and others safe from threats of severe food insecurity, the report recommends that the city develop community food storage plans with community leaders to ensure food is accessible by foot in all neighbourhoods in emergencies. This strategy aims to ensure more reliable access to food, particularly for food-insecure households who may be unable to purchase and store emergency food supplies in their home.

Another recommendation focuses on strategies to provide food assistance that is safe for clients with special dietary needs including health-related requirements, allergies and cultural requirements. FAO in Baltimore provide millions of meals each year to those in need. Yet most FAO are unable to control the donations they receive and cannot meet the unique dietary needs of all clients. This is particularly true in times of emergency, when FAO are focused on rapid mass distribution of food. In the words of one FAO employee:

‘Things like formula … that is not something that is typically available during disaster. It’s harder to find generally and there’s only a couple of places that do it, and that is very dangerous to me.’ (FAO1)

To address this concern, the report recommends that those responsible for emergency preparedness at the city and organization levels consider the needs of people with special dietary requirements in the planning process to ensure they can access safe foods in moments of crisis.

#### Trustworthiness and transparency

Because public policy has been used at times to oppress vulnerable communities (e.g. redlining^(^ ^40^ ^)^ and voter identification laws^(^ ^41^ ^)^), it is critical that the goals of trauma-informed policy and the procedures by which those goals will be attained are transparent^(^ ^32^ ^)^. Our report clearly lays out its goals, the rationale for each goal and the steps it recommends to achieve those goals.

Interviews indicated a pervasive feeling of distrust towards government among some community residents, the cumulative product of generations of marginalization and mistreatment. In the words of one community leader:

‘I think people generally in these kinds of communities are distrustful, and they’ve been burned a lot institutionally. Because of people’s dealing with addiction and poverty, people are less trusting too, because they’ve had lots of violations sometimes.’ (CL1)

To begin to heal wounds and build trust, the report recommends several strategies to encourage stronger relationships between community members and city institutions. For example, the report suggests including community members in all stages of development and implementation of the final Food System Resilience Plan. It also suggests the city establish long-term working relationships with trusted community leaders and collaborate with them on all future efforts to address food insecurity.

In the spirit of greater transparency, the report also recommends the city develop a communication and outreach campaign with information about the steps households can take to prepare for emergencies. The campaign would use trusted channels of communication recommended by interviewees, including Maryland 2-1-1 (a free social services resource hotline), local radio and faith leaders.

#### Collaboration and peer support

Collaboration with the policy’s target population and prioritization of indigenous knowledge are hallmarks of trauma-informed policy^(^ ^32^ ^)^. Interviewees expressed concern that often the community is only made aware of government plans once they are already finalized. One FAO employee explained that she hopes to see this change:

‘I would just want the community to be involved in all aspects. Not to be told here is your plan.’ (FAO2)

Our stakeholder interviews represent one part of involving the community from the outset. While limited resources prevented broad outreach at this phase in the process, we did seek input from a wider section of the Baltimore community by conducting outreach activities at two community events. We also sought input on the project at several city meetings convening representatives of Baltimore organizations involved in the food system. Interview and event participants provided information that helped us understand the strengths and weaknesses of the Baltimore food system and offered recommendations for how to improve local food security; this information formed the basis of our report, defining both policy goals and strategies to achieve those goals. For example, participants helped researchers identify key community institutions and common methods of communication used by residents. Recognizing the important role that institutions such as churches play in bringing people together and disseminating information throughout the community, the report recommends using these trusted organizations, among others, as central drop-off points for resources during emergencies.

The City of Baltimore plans to build on the report to develop the final Food System Resilience Plan. Community input will play an even greater role in that process.

#### Empowerment

Trauma-informed policy should empower the community both through the policy formulation process and the policy’s outcomes^(^ ^32^ ^)^. We sought to provide those who are affected by food insecurity with a voice in the policy-making process. As described above, many policy recommendations in the report were proposed by community members, and the city will continue to solicit community input and feedback as it develops the final plan.

In Baltimore, community-based organizations (CBO), which include FAO and other local organizations that provide social services, play an important role in representing and serving the needs of the people. Several interviewees explained that the city could most effectively tackle food insecurity and empower residents by lending support to the ongoing efforts of CBO. In the words of one FAO employee:

‘I would just return to talking to people like [influential community leader], people who already engaged in the solutions on the ground and know the communities best, and making sure that they set the direction for what’s needed and not duplicating efforts, but also not coming in and telling them what’s needed, but really listening.’ (FAO3)

Thus, several of the report recommendations focus on strengthening the capacity of CBO and the working relationship between CBO and food policy staff within city government. For example, the report proposes the city government provide technical assistance and resources to CBO to support their ongoing work to address food insecurity and to support emergency response planning and training. Additionally, many of the policy recommendations will be implemented in partnership with CBO.

#### Choice

Trauma-informed policy seeks to preserve people’s abilities to make choices freely and maintain a feeling of control^(^ ^32^ ^)^. When people experience food insecurity, their ability to exercise a fundamental freedom – deciding what to eat – is restricted. Their food choices may be limited by economic constraints (e.g. inability to afford cost of food) or physical constraints (e.g. no supermarkets in their neighbourhood). In the face of hazards, choice may be further limited. For example, a labour shortage caused by a pandemic may drive up food prices or flooding may close roads to supermarkets. Food insecurity especially restricts options available to those with special dietary needs, who have limited choices even in the best of circumstances.

Interviews indicated that during past citywide emergencies, food choices have been curtailed. One older community leader described her experience during recent adverse weather events; she had enough food to make it through the event, but her options were severely limited:

‘Usually because we’re older … I might do with what I have, but here I won’t go hungry. It may not be what I want, but I’ll have something. I think for me, being a community leader for so long, that’s kind of what I see. We may not have what we want, but we have something.’ (CL4)

In the report, we recommend policies to remove barriers to choice faced by people experiencing chronic or acute food insecurity. For example, for the nearly one-third of Baltimore residents who do not have vehicles, getting to a supermarket can be a challenge^(^ ^31^ ^)^. While corner stores are more common in low-income neighbourhoods without supermarkets, most offer limited or no produce and may not be a primary food source. We recommend the public transportation system be redesigned with food access in mind to improve supermarket accessibility and the food choices residents have.

Another recommendation focuses on preserving choice in case of an electrical power outage. Outages, which can occur independently or in conjunction with weather-related hazards, can disrupt refrigeration capabilities in homes and stores. To help retailers maintain their inventories, we recommend that the city offers retailers financial incentives to purchase backup systems such as generators or energy-efficient refrigerators. By preserving inventory at food retailers, we can preserve choices available to consumers.

#### Intersectionality

Trauma-informed policies must consider intersectionality, defined by Collins as ‘an analysis claiming that systems of race, social class, gender, sexuality, ethnicity, nation, and age form mutually constructing features of social organization’^(^ ^32^ ^,^ ^42^ ^)^. Awareness of intersectionality allows for an understanding of the relationship between an individual’s combination of identity characteristics and experiences of discrimination, privilege and human rights violations.

Intersectional policies must address historical trauma, defined as ‘cumulative emotional and psychological wounding across generations … which emanates from massive group trauma’^(^ ^43^ ^)^. Communities of colour in the USA have experienced a painful history of slavery and legalized discrimination and continue today to encounter pervasive structural and interpersonal racism. Over more than 150 years, African-Americans, who make up 63 % of the Baltimore population^(^ ^44^ ^)^, have experienced trauma in the form of land loss–stripped of land through government-sanctioned takings, or driven from it through intimidation and violence^(^ ^45^ ^)^.

Two community leaders discussed the importance of food sovereignty–the right of the people to define their own food and agriculture systems^(^ ^46^ ^)^–in addressing food insecurity and the need to reconnect African-Americans in Baltimore with land. In the words of one:

‘Personally, coming from a–I think a strong people … I don’t want nobody giving me something. If I wanted to grow for myself, I want a secure land. Land is important. People not having, again, control of land. Community control of land. That’s a crisis in and of itself.’ (CL2)

To help address this historical trauma, the report includes recommendations to support community-led efforts to engage in urban farming and community gardening. Participation in community-led agriculture could help connect participants to land and enable them to make their own decisions about the production, distribution and consumption of a portion of their food.

Intersectional policies should also work to address disparities as close to the root as possible, actively seeking to address the structural conditions linked to food insecurity such as poverty, unemployment and racism, which have for generations disempowered and disenfranchised vulnerable communities. Several of the policies we recommend in the report attempt to tackle these upstream factors. For example, one recommendation focuses on creating a more resilient Baltimore food business environment by encouraging diversity in food store ownership, size and type. To achieve this, we suggest implementing policies that support minority-owned and neighbourhood-owned new businesses and developing a range of financial and technical resources specific to the unique challenges storeowners face related to language, access to capital and educational attainment.

## Discussion

In creating the *Baltimore Food System Resilience Advisory Report*, we found that a trauma-informed approach is a valuable tool to guide development of food policy. The tenets of the trauma-informed framework are similar in key ways to several other well-known social justice-oriented models of policy development such as Rapp *et al*.’s model of strengths-based social policy analysis^(^ ^33^ ^)^, Sabatier’s advocacy coalition framework^(^ ^47^ ^)^ and the UN human rights-based approach^(^ ^48^ ^)^. For example, all four frameworks emphasize inclusion and collaboration^(^ ^32^ ^,^ ^33^ ^,^ ^47^ ^,^ ^48^ ^)^, and the trauma-informed and strengths-based frameworks both stress the importance of providing meaningful choice and place the onus of achieving the policy goal on political institutions and social systems rather than on the target population^(^ ^32^ ^,^ ^33^ ^)^.

Beyond these and other commonalities, however, the trauma-informed framework differs from others both in approach and outcomes. Bowen and Murshid’s approach is distinctly suited to developing policies to address social problems related to trauma, such as food insecurity. One key difference is that Bowen and Mushid’s framework is guided by a commitment to recognizing experiences of trauma, promoting healing and preventing re-traumatization, through both policy development and policy outcomes^(^ ^32^ ^)^. To achieve these aims, the framework promotes policies that prioritize the physical and emotional safety of the target population, and foster trustworthiness and transparency throughout the policy formulation process. Other frameworks that do not overtly prioritize safety, trustworthiness or transparency may unintentionally contribute to further trauma or lead to reduced effectiveness.

For example, the advocacy coalition framework focuses on building coalitions that can include members of the policy’s target population^(^ ^47^ ^)^. The framework could be considered trauma-neutral; it does not explicitly consider how participants may have experienced historical trauma at the hands of government or other social systems, and how they may thus lack trust in these institutions. Accordingly, while those using the advocacy coalition framework may be equally committed to building strong social policy as those using trauma-informed approaches, they may be less focused on empowering participants, promoting trust, recognizing long-term sequelae of traumatic histories and working purposefully to minimize future traumatizing experiences.

Additionally, unlike many other policy development frameworks, the trauma-informed approach places strong emphasis on directing attention and resources to address upstream determinants of health^(^ ^32^ ^)^. This approach ensures that polices address causes of trauma as close to the root as possible. Finally, the trauma-informed approach promotes policy solutions that intentionally target groups that have faced traumatic experiences, reflecting the reality that trauma is not equally distributed throughout the population^(^ ^32^ ^)^. Thus, the trauma-informed approach argues for providing resources and opportunities tailored specifically for communities that have experienced trauma (for example, creating central locations with stockpiles of food in vulnerable communities in advance of storms). This approach is distinct from the strengths-based framework’s argument that social policy should be designed to foster integration and ensure equal membership in society through equal access to resources, opportunities and locations for all people, and its argument against creating separate and ‘segregated resources’^(^ ^47^ ^)^.

### Improvements to our process and report

Bowen and Murshid write that policy is fraught with compromise and that any single policy is unlikely to reflect all the principles of trauma-informed policy^(^ ^32^ ^)^. Instead, the framework ‘provides a conceptual ideal whose aspects policies on the ground may reflect to varying degrees’^(^ ^32^ ^)^. While we sought to incorporate all the principles in the *Baltimore Food System Resilience Advisory Report*, we recognize that there are several ways we could have improved both our process and our final report.

First, staff and financial constraints limited our ability to solicit input from a broad array of community members. Although we interviewed community leaders who have a clear understanding of the challenges faced by members of their community and were able to describe their experiences, future work should seek to also include community members who directly experience food insecurity. Further, the community leaders included in the study represented neighbourhood associations and religious organizations; additional insight regarding the unique challenges faced by specific vulnerable populations could have been gained through interviews with a wider variety of community leaders (e.g. youth leaders or leaders in the disability community). Additionally, at our community outreach events, we were unable to reach those who were too busy to attend the outreach events or did not hear about them. These individuals – potentially those who work multiple jobs, those with significant family obligations and those are not plugged into neighbourhood channels of communication – are among the most vulnerable to food system hazards. Their insights are critical to the planning process. Fortunately, there is considerable opportunity to seek broader input. City officials will conduct additional and more direct community outreach as they develop the final resilience plan, all while maintaining the trauma-informed approach. The city plans to employ and further expand on methods it has used in the past to solicit community input; past methods have included creating an advisory panel of residents and other stakeholders, hosting community meeting events and sending representatives to neighbourhood association meetings^(^ ^28^ ^)^.

Finally, while we did attempt to address some of the upstream, structural determinants of food insecurity, because of the focused nature of the report, we were unable to adequately address some important determinants, such as land ownership and racial discrimination. These topics are addressed in other city plans, including the Baltimore City Health Department’s Healthy Baltimore 2020 strategic blueprint, which focuses on addressing upstream determinants of health disparities^(^ ^36^ ^)^, and the Baltimore Food Policy Initiative has committed to using a racial equity lens in its work on these issues.

### Challenges of trauma-informed policy development

There are several challenges associated with using a fully trauma-informed approach to developing social policy. Creation of trauma-informed social policy requires involvement of affected communities throughout policy development and implementation. Bringing affected community members to the table can be difficult given the many demands on time, but the challenge becomes especially acute when developing policies that target stigmatized issues such as reliance on food assistance, or when seeking participation from communities in which many adults work long or variable hours, may experience chaotic living situations, and may lack childcare and transportation to attend meetings.

Communities that have experienced trauma may also be particularly distrustful of government institutions and reluctant to participate in discussions on policy development. Policy developers must build trust within the community, including by working with trusted existing organizations. They also must avoid putting participants in situations that could expose them to further trauma.

Soliciting opinions from a broad, diverse group of community members is time- and resource-intensive. Funder outreach and education is needed to increase availability of funding for this type of community-participatory work.

As cities around the world consider their approach to improving food system resilience, there is a need to incorporate elements of a trauma-informed approach. Using this lens to develop the *Baltimore Food System Resilience Advisory Report* helped ensure policy recommendations were community-informed and designed to minimize the traumatic impact of food insecurity. Future research is needed to examine how the principles of trauma-informed policy are being incorporated into policy development in other areas, and to evaluate the implementation and impact of trauma-informed social policies.
